# Adapting water resource systems to a changing future: challenges for UK hydrology in the 21st century

**DOI:** 10.1098/rsta.2024.0278

**Published:** 2025-07-31

**Authors:** Jim W. Hall, Anna Murgatroyd

**Affiliations:** ^1^ Environmental Change Institute, University of Oxford, Oxford, UK; ^2^ Newcastle University, Newcastle upon Tyne, Tyne and Wear, UK

**Keywords:** hydrology, risk, uncertainty, decisions, modelling, climate change

## Abstract

The aquatic environment globally is under enormous pressure. People responsible for managing and regulating water resources face very difficult decisions about how to allocate water, restore the natural environment and use their scarce financial resources. Their decisions will depend upon challenging scientific questions about how catchments and river basins are going to change in the future and how they will respond to combinations of interventions at a range of different spatial scales. We contend that hydrological science in the UK is not yet, or in some instances even near to, providing the evidence that is needed to manage the quantity and quality of water resources in the 21^st^ century, given the scale and complexity of interventions that are now being considered and the uncertainty in catchment response. In some respects, hydrology has been exemplary in the decision-orientation of much of the research in the field. Enhancements in observation (especially Earth Observation), combined with modern data-driven methods are having a transformative impact on hydrology. But we will argue to tackle the water challenges for the UK in the 21st century requires further fundamental improvements in the capacity to model catchment processes and the impacts of a wide range of management interventions.

This article is part of the Royal Society Science+ meeting issue ‘Hydrology in the 21st century: challenges in science, to policy and practice’.

## 1. Introduction

Recognition of the importance of water has never been greater. News of devastating floods and droughts—the latter often also combined with extreme heat and wildfires—arrives with alarming frequency. Concern about water quality, in drinking water and in the environment, is also growing and sometimes hits the political headlines, as they have done in the UK over the last couple of years, with blame pinned on those with responsibility for ensuring water quality.

In systematic analyses, water-related risks are consistently of growing concern. The World Economic Forum’s 2024 Global Risks Report [1] ranked extreme weather events (which includes floods and droughts, as well storms, extreme heat, wildfires, etc.) as the second highest concern over the next 2 years and the top concern over the next 10 years. Critical changes to the Earth system (including the hydrological cycle and shifts in ocean-atmosphere phenomena including the monsoon and the El Nino Southern Oscillation), natural resource shortages and pollution all also appear in the top 10 risks, as does biodiversity loss and ecosystem collapse which includes aquatic ecosystems. In addition to such physical threats, the United Nations World Water Development Report 2024 [2] highlighted that tensions over water are exacerbating conflicts worldwide. In Africa, for example, 19 of 22 states studied for the UN 2024 report suffered from water scarcity, many of which rely on transboundary water resources with poorly formalized interstate cooperation.

The Global Commission on the Economics of Water issued its Call to Collective Action in March 2023 [3], in which it identified syndromes which will be all too familiar to water professionals: perverse incentives (in particular for inefficient water use in agriculture), under-pricing of water, and under-valuation of environmental externalities. However, scientific hydrology is not particularly visible in any of these analyses of water-related risks. The presented solutions are governance-related and economic—not surprisingly as the Global Commission was on the economics of water. Indeed, it is reasonable to argue that the water crisis is a crisis of water governance [4].

In the UK, extreme weather events and changes to long-term climate conditions threaten water resource systems. For example, the 2023 edition of the UK’s National Risk Register [5] categorizes flooding (river, coastal, surface water flooding) as being the most significant natural hazard. The UK’s third Climate Change Risk Assessment [6] identified flooding, droughts and impacts on the natural environment as being among the foremost risks facing the UK. Though awareness of these risks is growing, and significant steps are being taken to adapt water resource systems to floods and droughts, it is not clear whether these efforts are going to be sufficient to meet increasing challenges in the future.

The need for improved scientific evidence to inform governance reforms and decisions is recognized by the global scientific community [7], but hardly seems to be a priority when policy-makers turn their attention to water. This may, in part, be due to the quality and robustness of hydrological science being taken for granted in policy and economic circles. However, we will argue that it may also be a consequence of hydrological scientists not being sufficiently focused on the evidence needs of decision-makers. This perspective piece builds on previous assessments of the state of hydrological research [8,9], to contend that existing hydrological science is not yet meeting the evidence needs required to manage the quantity and quality of UK water resources in the 21st century. We advocate for forward-looking hydrological modelling that supports decision-oriented analysis and optimization, and provides evidence for decision-makers on a range of timescales for diverse water management applications.

## 2. Categories of water decision-making

To begin exploring a possible disconnect between scientific hydrology and the needs of decision-makers, we define categories of decision that most commonly confront decision-makers who are responsible for the aquatic environment in the UK:

(1) *Environmental regulation (including water allocation*): As use of water entails many environmental externalities, there must be a significant role for government in regulating the quantities of water that are withdrawn from water bodies for different users, and the quality of effluent discharges to the environment. Setting and monitoring these allocation and environmental limits requires scientific insight into the status and resilience of the aquatic environment.
(2) *Subsidies and incentives*: In some contexts, regulation alone is insufficient to achieve desired environmental outcomes, when these involve changing the behaviours of actors like farmers, or enabling nature conservation organizations, which can require payments or subsidies. This raises a multitude of questions for decision-makers about how financial instruments should be designed, how much funding is required, and for how long.
(3) *Investment planning and financial regulation*: Human civilizations have always depended upon water infrastructure, which is capital-intensive and long-lived. Deciding what storage, transfer, distribution and treatment infrastructure to invest in is one of the most challenging problems facing decision-makers in a changing future. It entails trade-offs between security of water supplies, the investments costs (which are ultimately borne by bill-payers and/or tax payers) and the environmental impacts (see point 1) [10–12]. In systems where water infrastructure is in private ownership (as it is in England) the decisions by owners of natural monopolies must be scrutinized by regulators who act in the interests of bill-payers.
(4) *System operation*: Water infrastructure requires near-constant operation and control, for example to regulate reservoir releases, manage water levels (e.g. for navigation, flood protection or agricultural drainage) and maintain water infrastructure assets. Each of these operational decisions needs to be informed (explicitly or implicitly) by information on the status of the water system, forecasts of the future, communication of needs and assessment of impacts.
(5) *Warning and emergency response*: Providing warning for floods or droughts through dissemination of hydrological forecasts can enable adaptive actions which can lessen the impacts. There have been continued advances in the quality and coverage for early warning systems, e.g. flood and drought forecasting and warning [13] and data-driven methods are showing ever-growing forecasting skill [14,15]. The understandable excitement about hydrological forecasting should not detract from the other, arguably more challenging, decision problems listed above.

This list of decisions that depend upon hydrological evidence can be ordered on a range of timescales: long-term (i.e. regulation, investment planning, subsidy/incentive design); operational (i.e. reservoir operation, water allocation, water level management, asset maintenance, subsidy allocation) and real-time (i.e. flood and drought forecasting and warning).

The distinguishing feature of longer-term decisions is that they need to incorporate consideration of longer-term changes that can be highly uncertain: climatic change, catchment changes, changes in demands for water and changing expectations for the environment. Each of these types of change represents a major research challenge for hydrological scientists and their colleagues in other disciplines. While climatic changes (which will be addressed next) can be taken as being exogenous to the catchment system (notwithstanding land-atmosphere feedbacks, which are significant in some contexts and scales), all of the others relate to the behaviours of actors within catchments, who drive changes in land cover, land management and infrastructure. These changes may all be influenced by the actions of regulators and other policy-makers, and may have intended or unintended consequences. Meanwhile demands for water will be shaped by economic (e.g. the price of agricultural products), technological (e.g. the demand for cooling water [16,17]) or behavioural (e.g. household water use practices) factors.

## 3. Addressing challenges to meeting the evidence needs of decision-makers

### (a) Climate projections to inform water systems adaptation

The possible impacts of climate change on water resources, including flooding, has been a particular focus of attention by the climate and hydrological science communities for more than three decades. Yet, the evidence available to decision-makers who have to plan for the future, from seasonal to multi-decadal timescales, is still fraught with problems. These include climate model biases compared with observations, and wide differences between the predictions of different climate models for variables of most relevance to hydrologists, i.e. precipitation and evapotranspiration (ET) [18].

In many respects, hydrological modellers have been some of the most demanding and advanced uses of climate model outputs. Because catchment response is a function of multiple inputs aggregated over space and time (sometimes with long memory), hydrologists call for spatial time series of climate variables, were early adopters of weather generators [19] and adapted their use to simulate transient scenarios that extend seamlessly from reconstructions of past counterfactuals of climate variability into future trajectories of change [20]. Because of the large uncertainties in climate projections of precipitation and ET, hydrologists have been at the forefront of exploration of uncertainty [21], and in particular the use of large ensembles of climate model outputs [22,23]. For example, techniques such as ‘decision-scaling’ [24] and ‘robust decision-making’ [25] have been designed to specifically account for climate model uncertainties in evaluations of water systems under climate stress.

There has been an inevitable tension between climate modellers’ desire for ever-higher model resolution to correctly resolve critical atmospheric processes and circulation patterns [26] and hydrologists’ need for large ensembles of spatial-temporal series of bias-corrected weather variables to drive probabilistic hydrological predictions. The UK’s next generation of climate projections are not yet planned (the last projections were published in 2018 [27]), so when they are being specified, there should be a strong hydrological voice at the table to ensure that the next projections contain:

— Large ensembles of high-resolution transient climate model runs, including representation of extreme events e.g. prolonged droughts;
— Evidence from multiple climate models from different global modelling centres;
— Retrospective credibility analysis of climate model outputs and clear presentation of estimates of model errors;
— Deeper use of observations (including historical and paleo data) to identify and explain trends and abrupt changes, and to transition plausibly from observed variability into future projections;
— Consideration of storylines e.g. circulation shifts that could drive significant shifts in precipitation [28];
— Coherent analysis of global changes and extremes (alongside UK climate projections), for analysis of international risks and interactions with the UK.

### (b) Hydrological modelling for evidence-based decision-making

Better evidence of future hydrological variability, from analysis of observations as well as from climate models, will be a significant step in informing climate adaptation, but that needs to be accompanied by much better understanding of river basin response to climate forcing and to other interventions.

To understand the challenges of droughts and water scarcity, there needs to be more complete analysis of how droughts materialize in surface water and groundwater bodies. This is particularly challenging for predicting the impacts of droughts of unprecedented severity. The UK’s National Infrastructure Commission has called for water supply systems in England to be resilient to droughts with a return period of 500 years [29]. First, that raises the challenge of how to estimate the severity (and associated uncertainties) of such extreme droughts, and second it raises the challenge of assessing system response to droughts of extreme severity beyond the observational record. Yield from many of England’s boreholes is constrained, at least at some times, by groundwater levels [30], yet exactly how groundwater will respond to a given combination of antecedent abstractions and dry weather needs to be more precisely understood.

Alongside the questions of quantity of water available in groundwater and surface water resources during droughts, is the question of how the aquatic environment may respond to extreme water scarcity and additional water withdrawals in these conditions. Ecosystems are naturally resilient to occasional droughts, but if they are in worse condition (e.g. due to pollution, over-abstraction, land drainage or channel modification) before they are impacted they will be less able to cope with and recover from periods of low flow [31]. Moreover, ecosystems are increasingly under chronic stress from climate change, which may be eroding their resilience. In Great Britain and Ireland, for example, small water bodies provide a suite of vital ecosystem services, yet they are highly vulnerable to climate, anthropomorphic and environmental change [32]. Understanding ecosystem resilience to droughts, and the extent to which they need to be restored to become more resilient, is a key question for aquatic ecologists. The answer will determine how much water can be sustainably withdrawn from the environment, especially during times of water scarcity, when calls to bend regulatory rules may be most shrill.

Analogous questions exist for decision makers and hydrologists that are concerned about water quality. In the UK, we do not yet have a complete picture of the point sources and diffuse pollutants that are being released into catchments, and their variation through time. While the processes of advection and diffusion of pollutants can be modelled, coupled with the biochemistry [33], uncertainties in parameterization of these processes, and spatial heterogeneity, means that our understanding of the capacity of water bodies to sustainably assimilate waste discharges, and the resilience of aquatic ecosystems to different concentrations of pollutants and flow regimes, is far from understood. This is even after years of monitoring in demonstration test catchments [34] and similar initiatives. The knowledge gaps are now becoming acute because of intense interest in interventions aimed to restore catchments—examples of which we will come to presently.

Finally, and arguably foremost given its position in the National Risk Register [5] and Climate Change Risk Assessment [6], flooding poses an intensifying challenge for hydrologists in the UK, as well as around the world. In the UK, flood hydrology is still based on the methods of the Flood Estimation Handbook (FEH) which was updated in 1999 [35]. Though in many respects FEH has served practitioners well [36], it has not been updated to reflect the extended observation record [37], improved statistical methods and the growing evidence of non-stationarity in hydrological observations [38]. In addition, machine learning methods are providing better methods for prediction in ungauged catchments [39], while decision-makers are asking more sophisticated questions, like the probability of flooding in multiple catchments at the same time [40]. The implications of uncertainty in the flood hydrology methods that are currently used in the design of billions of pounds-worth of flood protection are not known, but could reasonably be expected to be significant.

### (c) An expanding set of catchment interventions

When faced with the challenges of floods, droughts and water scarcity, harmful water quality and deteriorated aquatic ecosystems, policy makers are increasingly recognizing that they may have to intervene in many different ways. In Britain, for the first time in more than 30 years, a number of large new reservoirs are being planned to help to assure the resilience of water supplies. An estimated £56 billion may need to be spent to address sewage releases from waste water treatment works [41]. Meanwhile, there is ever-growing interest in the role of nature-based solutions, including natural flood management (NFM) and measures to mitigate diffuse pollution. The Environment Agency has already committed £25 million for NFM projects. In towns and cities, there is a growing call for the use of sustainable drainage systems (SuDS), which may need to be retrofitted into the urban fabric as well as being mandated for new developments [42]. Some challenges require an urgent response, but in most instances it is recognized that a long-term adaptive plan is required, which sequences a combination of different interventions.

There is widespread recognition of the significance of uncertainty and the need for a risk-based approach to developing adaptation plans [43]. This means that even for systems (like conventional urban drainage) where established design methods exist, they need to be reconfigured to explore the wide range of conditions that risk-based approaches imply. In many other instances there is not good scientific understanding of how proposed interventions perform. This is particularly true for NFM, where despite a proliferation of model studies [44] there is still a dearth of empirical observations in controlled conditions i.e. with a sufficiently long record of pre-intervention flood events, or carefully paired control sites [45]. There is evidence of effects of NFM in reducing flood peaks in small floods in small catchments, but the evidence for large floods is less compelling (as one would expect from a physical perspective) and empirical evidence from large catchments is absent. It is therefore imperative that hydrologists avoid overselling NFM by clearly communicating the limitations of potential interventions.

Recognizing the scale of challenges being faced in many catchments, the response is that ‘we need to do it all’; in other words, adopt ambitious combinations of interventions, possibly including land use, land management, channel restoration and floodplain modification, storage on a range of scales, transfers, aquifer recharge and a variety of urban interventions. Some of these interventions may be small and local, but they may be applied in multiple and different ways in various locations across the catchment. So hydrologists are being asked to quantify the aggregate effects of these multiple interventions, and to estimate their effect on catchment behaviours. Moreover, we wish to know the performance of these interventions in a very wide variety of conditions: floods and droughts not only of different return periods, but also different spatial patterns and sequences, as this will influence the way these events are processed in the catchment. We need to know how this performance may change in the future, and how it may be adapted should different scenarios materialize.

While we have discussed these challenges at the catchment scale, funding is allocated across several catchments and regulations/legislation are enacted at national scales. So not only is there a need to target and quantify many catchment interventions, but there is also a need to prioritize and monitor performance across multiple catchments at national scales.

### (d) Challenges for modellers of catchment response

This set of needs from decision-makers—to design, optimize and adapt a multitude of catchment interventions, and understand their performance at a range of scales—have arguably always existed. But hydrological capabilities in the past were so constrained that perhaps it was accepted that there was not a complete model-based solution. Yet since this issue of the *Phil Trans* is seeking to explore hydrology in the 21st century, we can set our sights higher.

The need to represent the effects of many small-scale new catchment interventions—for which there is not directly relevant empirical evidence of their performance—points inevitably to the need for physically based hydrological modelling, which numerically represents the effects of hydrological processes like infiltration, soil moisture, sub-surface flow, runoff, channel flow and sediment/nutrient dynamics. It also implies the need for fine enough spatial resolution to represent intervention in a not-too-abstract way, while avoiding common pitfalls of high-resolution modelling such as over-parameterization and high calibration uncertainty. In observation-rich countries such as the UK, with its long hydro-meteorological records, this issue can be navigated more easily than in data-poor countries. Moreover, rapid advances in Earth Observation have supplemented the data record in all contexts, though there are limitations to the hydrological parameters that are observable from space. When combined with advances in terrestrial observation (e.g. with cheap video sensors) we are observing impressive advances in data-driven hydrological modelling [14]. We can also anticipate advances in emulation of physically based models to build credible hyper-resolution models which capture physical processes [46,47]. However, the parameterization of aspects of these models that are hard to observe will continue to be a challenge until there are further breakthroughs in observation and data assimilation.

Of course, physically based hydrological modelling is not new. The physical equations of hydrological processes have been known for a long time and have been implemented on many occasions. The critiques are also well-known—that grid-scale parameterizations do not represent the same quantities that can be physically measured in nature, and that heterogeneity renders parameter estimation practically impossible in highly parameterized models [48]. Yet computational model studies have credibly reproduced the observed performance of catchment interventions, like storage ponds [49] and channel restoration [50] in the real world. This builds confidence that large numbers of such interventions could be modelled at large scales. Some studies have endeavoured to do that [51] though, while the 1 km scale of resolution is reasonable to represent widespread changes in land cover (e.g. afforestation), it is inappropriate to model smaller-scale interventions. Even for tests of land use change, it is hard to see how land use changes in upland catchments—on hillslopes and riparian zones—can be credibly represented at such coarse scale. Some of the questions that are being posed, like the effects of natural flood management interventions, agricultural practices to manage diffuse pollution, and wetland restoration on aquifer recharge, all imply a high level of physical representation. To represent the combinations of policy interventions that are now being proposed requires higher resolution modelling everywhere, which needs to be tested in a full range of spatial-temporal forcing conditions.

Another instance in which breakthroughs in observation and modelling are required (even though the physics has been known for a very long time) is in groundwater–surface water interactions, and groundwater response [52]. We do not yet have the ability to predict the reliability of groundwater resources to extreme droughts, like the 1:500 year droughts that the water industry is now planning for, when water level and yield response goes outside observed ranges and may be highly nonlinear. Realistically representing these processes is fundamental to predicting the resilience of groundwater-dependent water supplies, and to science-based management of England’s unique chalk streams.

It is well recognized that the bathing water crisis in Britain is a combination of pollution from agriculture (which ranges from diffuse runoff from pastures and arable fields, to some heavily loaded sources at intensive animal rearing sites), combined sewage overflow (CSO) spills, and, as was highlighted in the recent Royal Academy of Engineering report, waste water treatment effluents [53]. The water industry has said that it will cost £56 billion to reduce sewage overflows. However, it is not clear whether the proposed investment represents the most cost-effective or sustainable response. To establish that would require highly resolved catchment systems modelling that represents the pollutant loads from different sources, their transport and biochemical processes through the catchment, and the vulnerability (over a range of timescales) of different ‘receptors’, e.g. aquatic species and human bathers. Such a system could then be used to optimize alternative interventions, from drainage infrastructure, to waste water treatment, to land use change, to incentivizing changed agricultural practices.

Some of the agricultural practices to reduce diffuse pollution include changing stocking densities, planting of shelter belts, modifying drainage ditches and changing manure spreading. We know that the in-river response of these interventions is very sensitive to local slope, soil and vegetation characteristics, as well as to the prevalent weather conditions. While to model these interventions we need to represent them at very small scales, we need answers on much larger scales: what will the impacts be on bathing water quality in downstream reaches of rivers, or neighbouring coastlines, of large numbers of small upstream interventions. Politicians are being persuaded that these interventions may be the solution, but the evidence does not yet exist. The policy is running ahead of the science, which sometimes it has to, but that underlines the need for rapid advances in science, which includes observations, fundamental scientific understanding and modelling.

### (e) Understanding the social dynamics of catchment response

Hydrologists have always recognized the role of human interference in catchments, and have become increasingly concerned with how this should be represented within their modelling systems. The simplest and most pragmatic approach is through a set of exogenous scenarios, which make assumptions about the salient aspects of human behaviour, such as household water consumption or irrigation water withdrawals. Yet such an approach is soon thwarted by the recognition that human agents are adaptive—they respond to what they observe is going on in a catchment or to factors (e.g. the weather) that are also driving catchment response. Water demand changes with the weather, and farmers make decisions based on their observations of soil moisture. There are dynamic interactions between actors at a range of scales—for example via markets for agricultural products, in which price expectations create incentives for farmers.

These dynamic interactions between the aquatic environment and human actors in catchments have enthralled a range of disciplines, including hydrologists, but also economists and sociologists. The sub-discipline of hydroeconomics [54] has its origins in the Harvard Water Programme of the 1960s and has continued to flourish with the development of partial equilibrium and agent-based models that simulate farmer behaviours, water use, farm yields and agricultural markets.

More recently, the field of sociohydrology was introduced as ‘a new science of people and water’ [55] and has spawned a proliferation of models of the dynamic relationship between the biophysical and socio-economic aspects of catchments, including some enlightening stylized models [56] and more realistic, and highly parameterized simulations, which can claim to have an element of empirical validity [57]. Sociohydrology’s bold goal to ‘make predictions’ [55] about the dynamics of coupled human-natural systems remains a challenge for interdisciplinary scientists in the 21st century. There are fundamental limits to the extent to which human behaviour is predictable (known as Shackle–Popper indeterminacy [58]), and even the more regular and predictable patterns of human behaviour are constrained by insufficient salient data from a range of different contexts. Further advances in our understanding and monitoring of socio-hydrological systems might be found from analysis of newspaper archives and tools such as Google Trends, which have been used to mine public sentiment and real-time occurrences of hydrologic extremes such as floods and drought [59]. Notwithstanding the profound limits on our capacity to predict the outcomes of coupled human–water systems [58], there is much more to learn about those dynamics, and new ways of collecting data about people, as well as catchments, from crowd sourcing to natural language processing, should improve our capacity to predict whole-catchment response.

### (f) Integrated modelling with decision-making

While socio-hydrology seeks to characterize the behaviour of human actors *within* the catchment system, decision analysis aims to inform choices between a set of alternative exogenous interventions *upon* catchments. These are difficult choices for two fundamental reasons. First, because there are multiple, to some extent incommensurate, objectives, usually including questions of economic productivity (e.g. hydropower, cooling water, water for industry, waterway navigation), social objectives (e.g. recreation, culture) and environment (biodiversity, landscapes, etc.). Second, the future behaviour of catchments, and hence their performance with respect to multiple objectives, is uncertain. The hydrological community has been at the forefront of developing and applying methods for dealing with both of those categories of problem: multi-objective, multi-criteria or multi-attribute decision problems; and problems of decision-making under uncertainty—as well as combining the two.

Methods for multi-attribute decision analysis have adopted multi-objective objective optimization techniques and applied them to a wide range of water management problems [60], including various instances of the nexus between water, food and energy [61,62]. A challenge is to properly translate the biophysical quantities that catchment models usually predict, into quantities of interest to decision-makers, often in monetary terms—though multi-attribute decision-making avoids the invidious task of having to monetize everything to reduce it to a single dimension.

**Figure 1.**
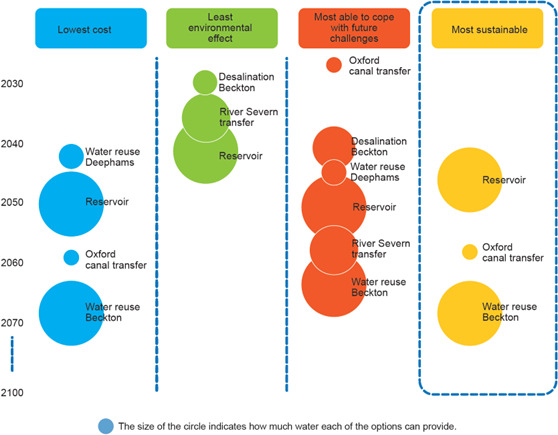
Example of decision analysis visualization used to evaluate supply-side interventions in Thames Water’s draft 2019 Water Resources Management Plan.

The concept of having to navigate trade-offs in water resource planning has been influential in practice. Figure 1 is taken from a draft version of Thames Water’s 2019 Water Resource Management plan, and illustrates the sequence of supply-side infrastructure investments that would be planned for the Thames system to secure water supplies in the future depending on three different priorities: least cost, least environmental effect, and ‘most able to cope with future challenges’, which roughly equates to the highest reliability of supply. The right-hand column represents a compromise solution that is deemed to be ‘most sustainable’. Continued improvement in the connection between catchment systems analysis, and decision tools like the one shown in figure 1, will enable better navigation of the difficult trade-offs that will be faced by decision-makers in the future.

Predicting future catchment performance is usually thought of as entailing two categories of uncertainty: aleatory uncertainties, which related to the random variability in catchment processes, most notably the weather, but also some of the variability in soils; and epistemic uncertainties, which relate to our lack of knowledge [63,64]. The field of stochastic hydrology for dealing with aleatory uncertainties was extensively elaborated in the latter part of the 20th century [65] and has demonstrable value in risk-based decision-making in hydrology [66]. It is now embarking on a new phase thanks to the application of machine learning. Probabilistic characterization of hydrological variability enables us to calculate risks, for example of flooding [63] or of water shortages [12]. Those risk calculations provide a direct way of trading off risk with cost to arrive at proportionate adaptation decisions [67].

Probabilistic risk estimates are themselves uncertain. Our lack of knowledge means that they contain epistemic uncertainties, which also have to be incorporated into decision-making. Epistemic uncertainties are more often associated with so-called ‘deep uncertainties’—situations in which it is not reasonable to characterize the uncertainty with probabilities. In that context, a variety of set-based methods have been developed and adopted [68], most notably robust decision-making (RDM) [69], but also dynamic adaptive policy pathways [70], decision scaling [71], info-gap [72,73] and imprecise probabilities [74]. Other epistemic uncertainties can exist within the hydrological modelling process itself (as well as other models e.g. climate models), where physical processes may be insufficiently represented. Sensitivity analyses designed to quantify model errors such that they can be incorporated in decision-making can help to navigate this uncertainty [75,76]. Underpinning all of these is the quest for relatively robust decisions—policies or interventions that perform acceptably well under a wide range of possible future conditions.

**Figure 2.**
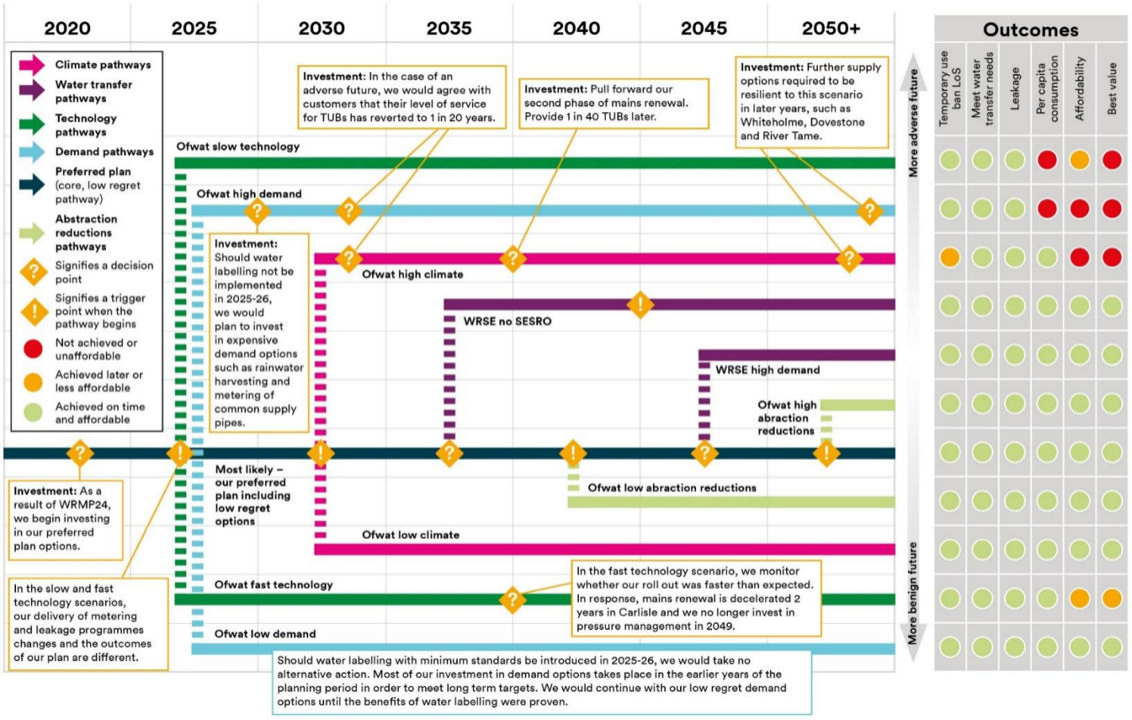
United Utilities adaptive plan for their Water Resource Management Plan 2024 [82].

When faced with uncertainty, it is well recognized that the capacity to adapt and change course can be of great value. Adaptable decisions are a way of achieving robustness. So, methods of sequential decision-making have also taken hold in water resources. In fact, water resource management has been one of the most widely cited examples of adaptive management [77]. Theoretical approaches to dealing with sequential decisions, including real options analysis [78], indicator-informed decisions [79] and dynamic programme [80] have been widely applied. The concept of dynamic adaptive pathways has gained a lot of traction with practitioners who are worried about uncertainty and committing too much too soon, and later regretting their decisions [81]. For example, figure 2 is from United Utilities Water Resource Management Plan, and illustrates the adoption, at least at a conceptual level, of adaptive management pathways, and also (on the right hand side of the diagram) a multi-attribute evaluation of decision alternatives.

This discussion underlines how methods for decision analysis are widely explored and adopted for water resources decisions. No doubt algorithms, computation and visualization will continue to improve. But at the kernel of all of these methods is a catchment simulator, which predicts catchment performance for a given combination of input variables. The greatest research effort is required to improve the fidelity of those simulations and the range of catchment variables and management interventions that they can represent.

## 4. Conclusions

In many respects, decision-makers have been well-served by hydrological science in the 20^th^ and early 21^st^ centuries. Hydrologists have engaged with the challenges facing decision-makers who have to navigate trade-offs between different goals and make decisions under conditions of deep uncertainty. They have developed risk-based methods that enable the trade-off between uncertain harmful impacts (such as floods and droughts) and the cost of reducing risks. Hydrological forecasting systems (which have not been a focus of this Perspectives paper) have continued to advance, helping to reduce some of the losses from extreme events. The advent of machine learning is helping to improve these systems still further.

However, as the pressures on catchments increase, and the urgency to find solutions to enhance their resilience and sustainability intensifies, new demands are being placed on hydrologists’ capacity to simulate hydrological processes and catchment response. This includes processes of peak flows and inundation; low flows, soil moisture and groundwater droughts; water quality; sediment transport and aquatic ecology. We have explained how greatly improved physically based modelling is needed in order to represent the increasingly complex and multi-scale management interventions that are being proposed within catchments and to evaluate them with respect to economic, societal and environmental objectives. We are realistic that there will inevitably be limitations in any model, but further improvements are required in the representation of uncertainty, so that models can be used with confidence as tools within robust decision-making processes. We expect that model improvements will be driven and enabled by improvements in gauging and sensing (including continued developments in Earth Observation), and the capacity for data assimilation and emulation (including with AI and machine learning). Such modelling advances would also enable the generation of multi-variate reanalyses for every catchment (i.e. realistic physically based reconstructions of what has happened in the past) which has proved to be tremendously valuable in climate science. Beyond model development, research efforts to improve the dissemination of observation data, forecasts and to strengthen governance structures will help to build resilience of vulnerable communities to hydrologic hazards in the UK and beyond. Sharing this research with less well-equipped nations that lack basic hydrological data, have limited technical capacities and weak governance is an equally pressing challenge to be faced by hydrologists in the 21st century.

Forward-looking modelling will improve the decision support platforms for what-if analysis and optimization, to provide evidence for decision-makers on a range of timescales: forecasting of floods, droughts and water quality incidents for a range of users; operational tools, that could be used for fine-tuning agricultural practices, urban water management (including discharges), water level management for environmental flow solutions, and for water allocation reservoir operation and transfers. Hydrological modelling to inform longer-term decisions, including infrastructure (flood protection, storage, transfers, waste water treatment, drainage), land use planning and agricultural policies, has existed for many years, and hydrologists have done well to adopt modern computer-based methods of decision analysis and visualization. But in the UK at least, we have not yet arrived at an integrated systems approach to catchment modelling that can explore the suite of options necessary to navigate catchments to a more sustainable destination.

## Data Availability

This article has no additional data.
